# Production and identification of two antifungal terpenoids from the *Posidonia oceanica* epiphytic Ascomycota *Mariannaea humicola* IG100

**DOI:** 10.1186/s12934-020-01445-7

**Published:** 2020-10-01

**Authors:** Lorenzo Botta, Raffaele Saladino, Paolo Barghini, Massimiliano Fenice, Marcella Pasqualetti

**Affiliations:** 1 Department of Ecological and Biological Sciences, University of Tuscia, Largo Università snc, 01100 Viterbo, Italy; 2 Laboratory of Applied Marine Microbiology (Conisma), University of Tuscia, Largo Università snc, 01100 Viterbo, Italy; 3 Laboratory of Ecology of Marine Fungi (Conisma), University of Tuscia, Largo Università snc, 01100 Viterbo, Italy

**Keywords:** *Mariannaea humicola*, marine fungi, antifungal activity, terpestacin, fungal xenicanes

## Abstract

**Background:**

Marine fungi are an important repository of bioactive molecules with great potential in different technological fields, the annual number of new compounds isolated from marine fungi is impressive and the general trend indicates that it is still on the rise. In this context, the antifungal and antimicrobial activity of the marine strain *Mariannaea humicola* IG100 was evaluated and two active terpenoids were isolated and characterized.

**Methods:**

Preliminary screening of activity of marine strain IG100 was carried out by agar plug diffusion methods against fungal (*Penicillium griseofulvum* TSF04) and bacterial (*Bacillus pumilus* KB66 and *Escherichia coli* JM109) strains. Subsequently, inhibition tests were done by using the cultural broth and the organic extract (ethyl acetate, EtOAc) by the agar well diffusion methods. The main active fractions were identified and tested for their antifungal activity against *P. griseofulvum* TSF04 in a 24 wells microplate at different concentrations (1000, 100, 10 and 1.0 µg/mL). Two active compounds were characterized and their relative MIC measured by the broth micro-dilution methods in a 96-well microplate against *Aspergillus flavus* IG133, *P. griseofulvum* TSF04, and *Trichoderma pleuroticola* IG137.

**Results:**

Marine strain IG100 presented significant antifungal activity associated with two active compounds, the terpenoids terperstacin **1** and 19-acetyl-4-hydroxydictyodiol **2.** Their MIC values were measured for *A. flavus* (MIC of 7.9 µg/mL and 31.3 µg/mL for **1** and **2**, respectively), *P. griseofulvum* (MIC of 25 µg/mL and 100 µg/mL for **1** and **2**, respectively) and *T. pleuroticola* (MIC > 500 µg/mL and 125 µg/mL for **1** and **2**, respectively). They showed a rather good fungistatic effect.

**Conclusions:**

In this study, the first marine strain of *M. humicola* (IG100) was investigated for the production of bioactive molecules. Strain IG100 produced significant amounts of two bioactive terpenoids, terperstacin **1** and 19-acetyl-4-hydroxydictyodiol **2**. The two compounds showed significant antifungal activities against *A. flavus* IG133, *T. pleuroticola* IG137 and *P. griseofulvum* TSF04. Compound **2** was identified for the first time in fungi.

## Introduction

Marine environments are sources of biological and chemical diversity, as well as a limitless resource of unexploited and unknown microorganisms. In addition, oceans are enormous repository of natural substances with applications in food and detergent industries, medical chemistry, biotechnology and biosensing [1–3]. To date, several drugs are derived from marine environments [4], and about 30.000 new bioactive molecules have been identified to asses a $5 billion global market [5, 6].

Nevertheless, the exploitation of bioactive molecules from marine origin is still scarce if compared to other natural environments [1, 7], especially in the case of marine fungi [8, 9]. In the last decades, the interest in marine and marine derived fungi has been growing due to their high efficacy in the synthesis of secondary metabolites [4, 5, 10–18]. These organisms are also promising sources of enzymes useful in different technological applications [19–21]. In this context, fungi isolated in highly stressing environments (i.e. from contaminated sites or hypersaline environments) have been studied and applied in the fine and specialty chemical fields, as well as in the production of biodiesel and bioremediation [9, 22–26]. The annual number of new compounds isolated from marine fungi is impressive and the general trend indicates that it is still on the rise [23]. The posidonia meadows represent the most important coastal ecosystem in subtidal shallow waters of the Mediterranean Sea [27, 28]. The seagrass *Posidonia oceanica* hosts a great variety of fungi associated to leaves, rhizomes, roots and matte. In particular, fungi related to the leaf district are characterized by a high content of tannic acids and represent an interesting source of new bioactive molecules [29]. A great heterogeneity of fungal colonisers from different posidonia meadows in Tyrrhenian Sea was reported [30–32], and their use in biotechnological applications is still increasing [20, 29]. *Mariannaea humicola*, belonging to Ascomycetes (Hypocreales, Nectriaceae), is generally recognised as a soil saprophytic fungus (ethimology: name refers to the soil substrate from which this fungus was isolated), [33]. Recently, the first marine strain of the species (*M. humicola* IG100) was isolated within an ecological study on the marine microbial biodiversity in a *P. oceanica* meadow [30]. Here we report the purification and isolation of two secondary metabolites of the terpenoid family from *M. humicola* IG100, characterized by a significant antifungal activity against *Penicillium griseofulvum* and *Aspergillus flavus*. The antimicrobial activity of these compounds was also evaluated against Gram negative and positive bacteria.

## Results and discussion

### Phylogenetic analysis of the fungus

**Fig. 1:**
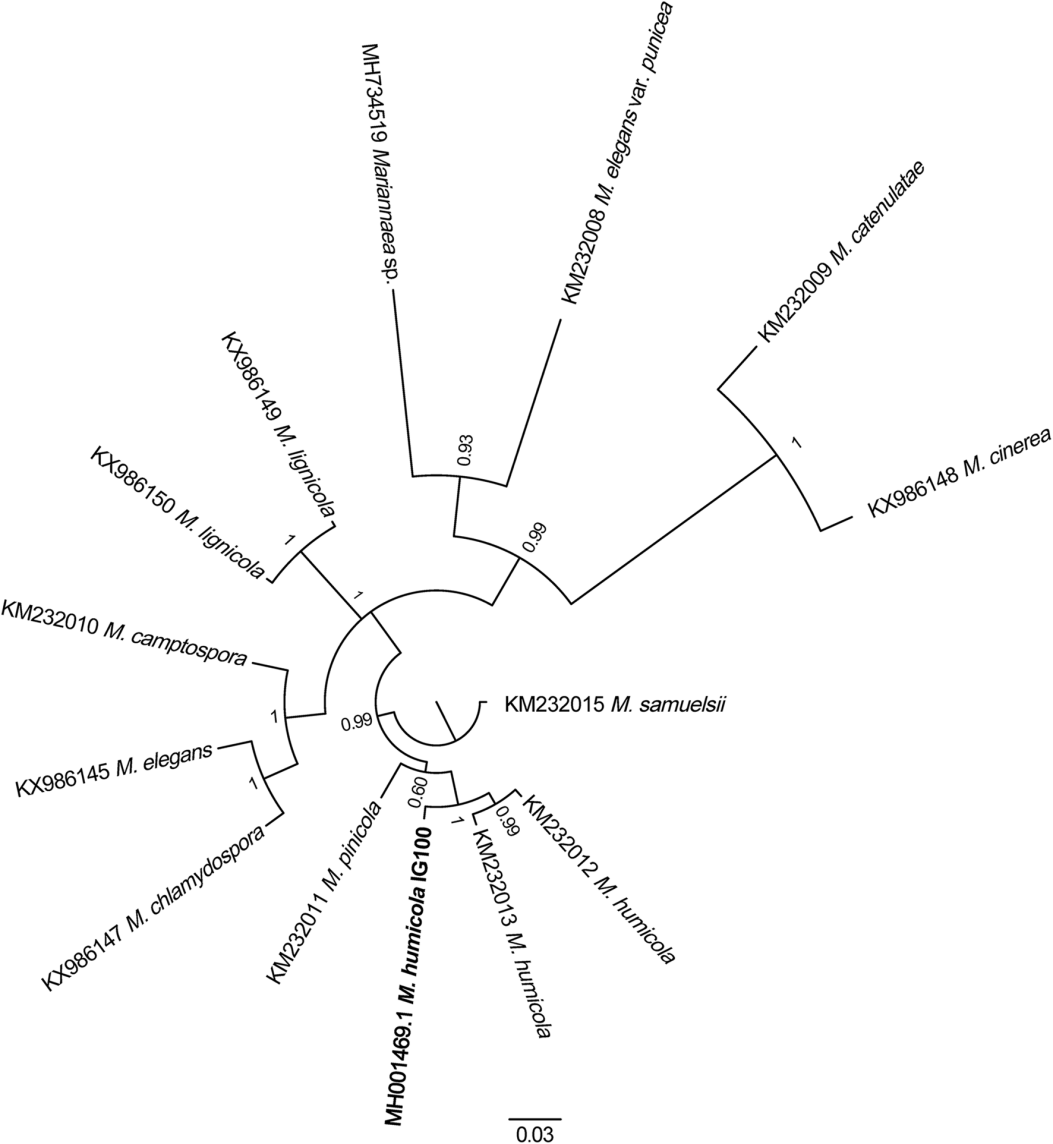
Bayesian phylogram based on the β-tubulin gene partial sequences for genus *Mariannaea*. Branch number indicates Bayesian Posterior Probability analysis (BPP)

The strain IG100 is well adapted to the sea conditions and its growth preferences permitted to considered this fungus as a marine strain of *M. humicola.* Actually, the strain is a facultative halophile (range of salinity tolerance 0–152% NaCl—optimum at 20%) with a sub-mesophilic behaviour, being able to grow in the range of 10–35 °C, with optimum at 25 °C [30]. The taxonomical identification was based on the ITS (GenBank accession number: MG976446) and β-tubulin gene (GenBank accession number: MH001469) markers [30]. In this study a phylogenetic analysis based on the β-tubulin gene was carried out within the *Mariannaea* genus using the GenBank database, which already include the IG100 strain (Fig. 1). All sequences relative to the *Mariannaea* species present in NCBI were considered. The phylogram clearly grouped IG 100 with two different strains of *M. humicola* with a sequence similarity of 98%. The phylogenetic analysis confirmed the previous taxonomical identification [30]. In addition, a certain degree of β-tubulin gene differentiation in the marine strain IG100 was observed with respect to other species of *M. humicola*, namely CBS 102,628 (KM232013, from decayed wood), and CBS 740.95 (KM232012, from soil). These data are in agreement with results of others that compared marine strains and their terrestrial counterparts [34, 35].

### Biological activity of strain IG100

**Fig. 2:**
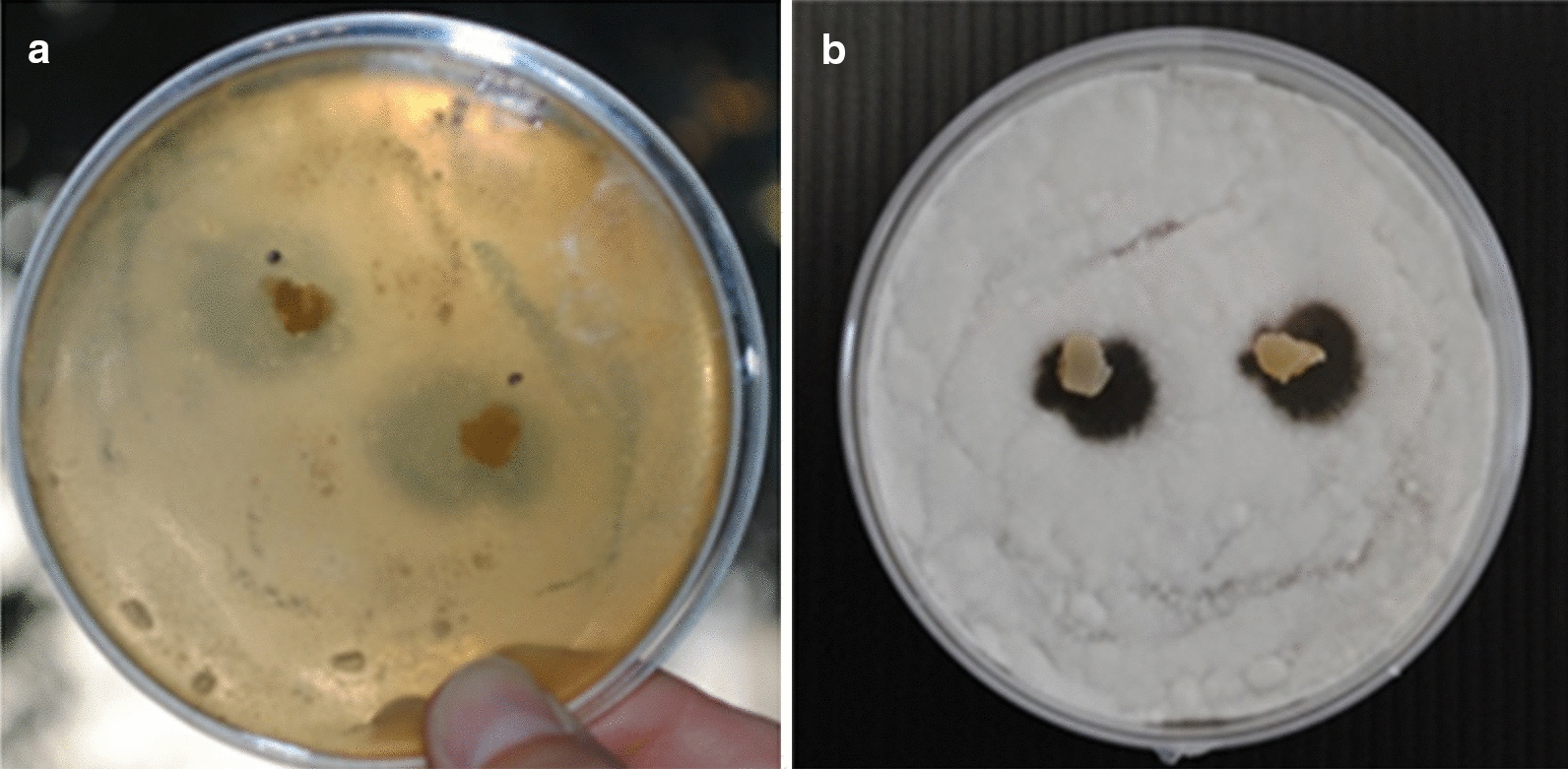
Preliminary agar plug screening (diffusion method) of *M. humicola* against *P. griseofulvum.* Panel a: activity at 48 h. Panel b: activity at 72 h

The preliminary plug screening [36] of strain IG100 was carried out using 12 days old agar plate cultures (MEAs) against *P. griseofulvum*, that is a common toxigenic postharvest fungal pathogen [37]. In addition, Gram positive and Gram negative bacteria (*Bacillus pumilus* and *Escherichia coli*, respectively) were tested (Table 1). The plugs of *M. humicola* IG100 revealed the presence of antifungal activity characterized by an inhibition area of 85 ± 5 mm^2^ at 48 h (Fig. 2a), and of 43 ± 3 mm^2^ at 72 h (Fig. 2b). The asymmetrical shape of the inhibition zone was due to the release of secondary metabolites at the fungal growing front. No activity was observed against both types of bacteria. On the basis of the activity observed in the preliminary test, the subsequent experiments have been carried out in liquid cultures (Malt saline medium) and the relative inhibition tests were done by using both the cultural broth alone and the crude organic extract (ethyl acetate, EtOAc) of the whole culture (12 days of cultivation). The broth activity was evaluated after 7, 14 and 21 days of growth by the well diffusion method [36] (Table 1), but no inhibition against the test organisms was recorded. By contrast, the organic extract (0.6 mg/mL, tested in the presence of 2% DMSO) after 12 days of cultivation showed a clear antibacterial and antifungal activity against *B. pumilus* and *P. griseofulvum*. No activity was observed against *E. coli*. In particular, the antifungal activity of the organic extract was 70–72% of the positive control at 48 h (0.05 mg/mL Myconazole). The antibacterial activity against *B. pumilus* was significantly lower than the antifungal one, with an inhibition area of 28–31% of the positive control (0.2 mg/mL Streptomycin) after 24 h of incubation (Table 1).

**Table 1:** Summary of preliminary screening test

	Agar plug diffusion method	Agar well diffusion method	
		Broth*	Extract
incubation time (h)	Linear inhibition (mm)	Inhibition to the control (%)	
	***P. griseofulvum***		
48	6.3±0.3	No effect	70–72
72	4.5±0.2	No effect	53–55
	***B. pumilus***		
24	No effect	No effect	28–31
48	No effect	No effect	18–22
	***E. coli***		
24	No effect	No effect	No effect
48	No effect	No effect	No effect

* Cultural broth at 21 days was reported as selected sample

### Identification of bioactive compounds and screening of antifungal activity

**Fig. 3:**
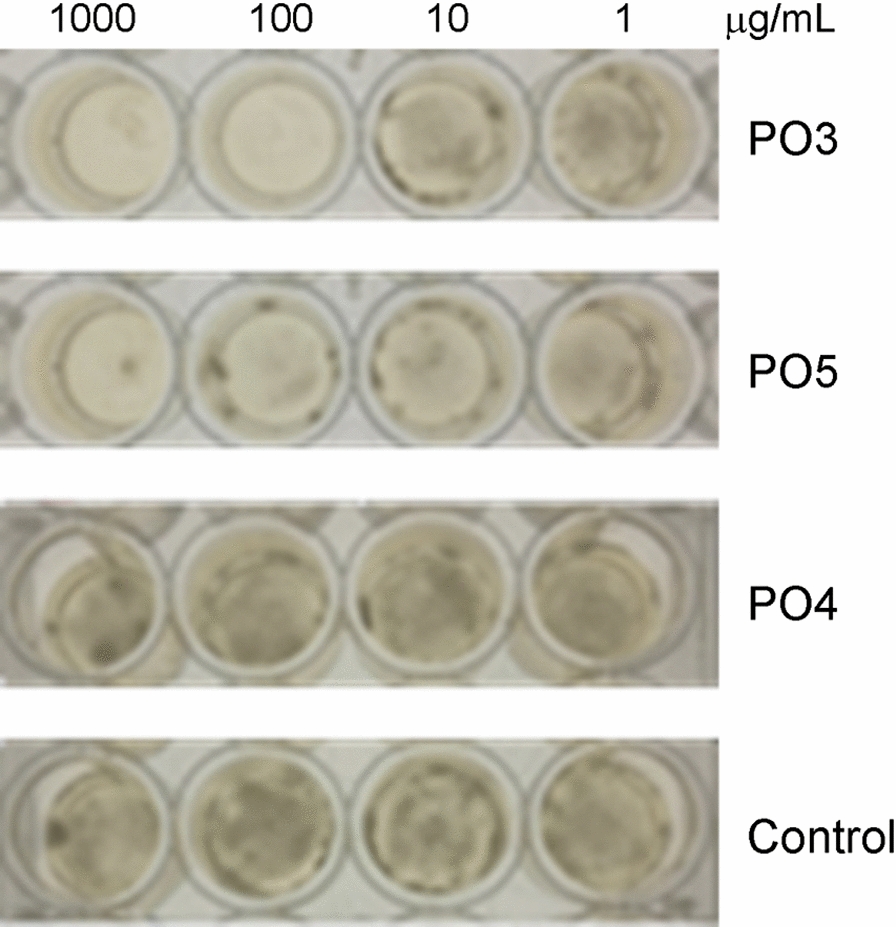
Antifungal activity of the identified purified compounds at different concentrations (1000, 100, 10 and 1.0 µg/mL) in crude extract against *P. griseofulvum*

Repeated cycles of flash-chromatography allowed to yield five major fractions (PO1–PO5) from the organic extract. The antifungal activity of PO1–PO5 against *P. griseofulvum* was preliminary tested in a 24 microwell plate at different concentrations (1000, 100, 10 and 1.0 µg/mL). A complete inhibition of the mycelial growth was observed for PO3 and PO5 at 100 and 1000 µg/mL, respectively, with a significant interference on the fungal growth until concentration of 1.0 µg/mL (Fig. 3). The other fractions showed only slight (PO1 and PO2) or no antifungal activity (as documented in Fig. 3 for PO4). Fractions PO3 and PO5 were further purified by semipreparative high performance liquid chromatography (HPLC) and the corresponding compounds were isolated and characterized.

### Structural characterization of active compounds

**Fig. 4:**
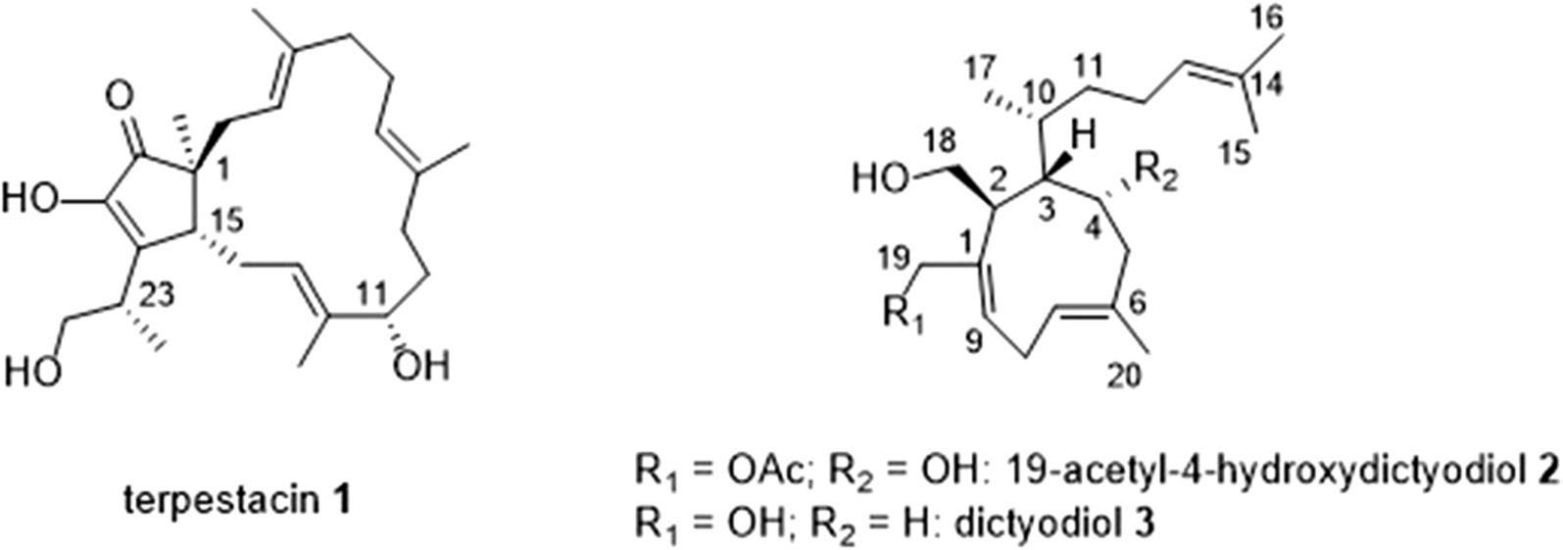
Chemical structure of terpestacin **1**, 19-acetyl-4-hydroxydictyodiol **2** and dictyodiol **3**

The isolated compounds were identified as terpestacin **1** (from PO3) and 19-acetyl-4-hydroxydictyodiol **2** (from PO5) (Fig. 4). Terpestacin **1**, obtained as a pale-yellow oil, was unambiguously identified by comparison with data previously reported [38]. The HRESIMS analysis yielded the expected chemical formula for **1** (C_25_H_38_O_4_), and the ^13^C NMR analysis showed all the expected carbon signals, corresponding to one carbonyl moiety (δ 208.0 ppm), six olefinic carbons (δ 138.3, 136.7, 133.1, 129.1, 124.5, 121.7 ppm), five methyl groups (δ 16.4, 15.8, 15.5, 14.6 10.7 ppm) and one oxy-methylene moiety (δ 66.3 ppm). In accordance with the proposed structure (Fig. 4), the ^1^H NMR analysis showed the typical signals for the sp^2^ hydrogens on carbons C-3, C-7, and C-13 at δ 5.41, 5.25, 5.14 ppm, respectively. In addition, the presence of a signal at δ 4.07 ppm, corresponding to the proton located on C-11, definitively confirmed the structure of **1** with respect to the possible epimer 11-epiterpestacin (not shown). ^1^H NMR signals of five methyl groups (δ 1.65, 1.64, 1.58, 1.30, and 1.01 ppm, respectively), and of two methines (δ 2.72 and 2.68 ppm), completed the assignment.

Terpestacin **1** was first isolated from the soil fungus *Arthrinium* sp. [39], and subsequently it was detected in several other fungi derived from saprotrophs, endobiotic symbionts (endophytes, gut of animals) and phytoparasites [40–43]. Among them, only two marine derived fungi were reported to produce **1**, *Fusarium proliferatum* a mangrove-derived endophytic fungus, and *Arthrinium* sp. isolated from the gut of a marine crab [41, 42]. Terpestacin-producing fungal species belong to the subphylum Pezizomycotina (Ascomycota) and are distributed into several classes (Sordariomycetes, Dothideomycetes, Leotiomycetes, Eurotiomycetes), orders (Hypocreales, Pleosporales, Thelebolales, Onygenales, Botryosphaeriales, Helotiales) and families (Nectriaceae, Apiosporaceae, Pleosporaceae, Thelebolaceae, Onygenaceae, Botryosphaeriaceae, Rutstroemiaceae). In addition, the biosynthetic gene cluster for terpestacin (*tpc*), identified from *Bipolaris maydis*, and from other homologous gene clusters, have been found in phytopathogenic strains [40]. Compound **1** is characterized by several biologic activities, including antiviral [39], anticancer [44, 45], antiangiogenic and cytotoxic effects (against several test cell lines and brine shrimps) [45, 46], and it was reported as a phytotoxic metabolite produced by pathogenic fungi in plants (*Neofusicoccus batangiarum* on *Opuntia ficus-indica*; *Rustroemia capillus-albis* on *Bromus tectorum*; *Bipolaris sorokoniana* on wheat and barley) [43, 47, 48]. The studies regarding the antifungal or mycotoxic activity of terpestacin are limited to only three phytopathogen fungal species (*A. brassicicola*, *B. cinerea* and *F. graminearum*). In these latter cases a hyphal growth reduction of the test organisms was observed as a possible consequence of an allelopathic action [49].

The 19-acetyl-4-hydroxydictyodiol **2** (xenicane-type diterpene) was obtained as a colourless oil and its structure was confirmed by comparison with data previously reported [50]. The molecular formula of C_22_H_36_O_4_ obtained by HRESIMS analysis was in accordance with the propose structure. IR and ^13^C NMR analyses showed the presence of a carbonyl function (1737 cm^− 1^ and δ 142.4 ppm, respectively). The presence of this carbonyl group is important to distinguish compound **2** from another similar xenicane-type diterpene, dictyodiol **3** (Fig. 4). In addition, ^13^C NMR spectrum highlighted the presence of all expected olefinic carbons (δ 135.2, 131.9, 130.0, 128.5, and 124.5 ppm, respectively), two oxy-methylenes (δ 67.0 and 60.8 ppm), one oxy-methine (δ 74.6 ppm), and one methyl carbon (δ 20.4 ppm). The ^1^H NMR confirmed the presence of the protons on the olefinic sp^2^ carbons C-7, C-9, and C-13 (δ 5.30, 5.82 and 5.07 ppm, respectively), and of the methyl groups in positions C-15, C-16, C-17 and C-20 (δ 1.60, 1.68, 1.02, and 1.97 ppm), respectively.

Xenicanes are a large class of marine diterpenoids featuring a cyclononane ring as a common structural denominator [51]. These compounds have been detected in several species of brown algae belonging to the genera *Dictyota* and *Dilophus* [51, 52] and could be involved in defensive processes, which greatly contributes to the successful survival and reproduction of the host in diverse marine environments. The xenicanes are characterized by antiproliferative, anti-inflammatory, and antifungal effects. Information on their *in vivo* activity is still lacking [53]. As far as we know, compound **2** was isolated previously only from *Dictyota plectens* [50] and it was never detected in fungi. Thus, the presence of **2** in brown algal extracts, which may be due to the release of the compound from algicolous fungi, cannot be completely ruled out.

### Minimal inhibitory concentration of the purified compounds

Considering the quite good antifungal activity observed in the preliminary analyses, a further biological assay was carried out in order to determine the minimal inhibitory concentration (MIC) of compounds **1** and **2** against *P. griseofulvum, A. flavus* and *T. pleuroticola*. The halotolerant fungi *A. flavus* and *T. pleuroticola* were considered for their ecological, agronomical, economical and clinical significance. *A. flavus* is the main agent of human allergic and bronchial aspergillosis of pulmonary infections in immunocompromised patients [54]. In addition, this species produces very harmful mycotoxins (aflatoxins, polyketide secondary metabolites that are potentially carcinogenic) in oil-rich seeds (corn, cotton, peanuts, hazelnuts and walnuts) [55]. *T. pleuroticola* is a mycoparasite causing severe green mould diseases in the commercial fungal genus *Pleurotus* [56].

**Fig. 5:**
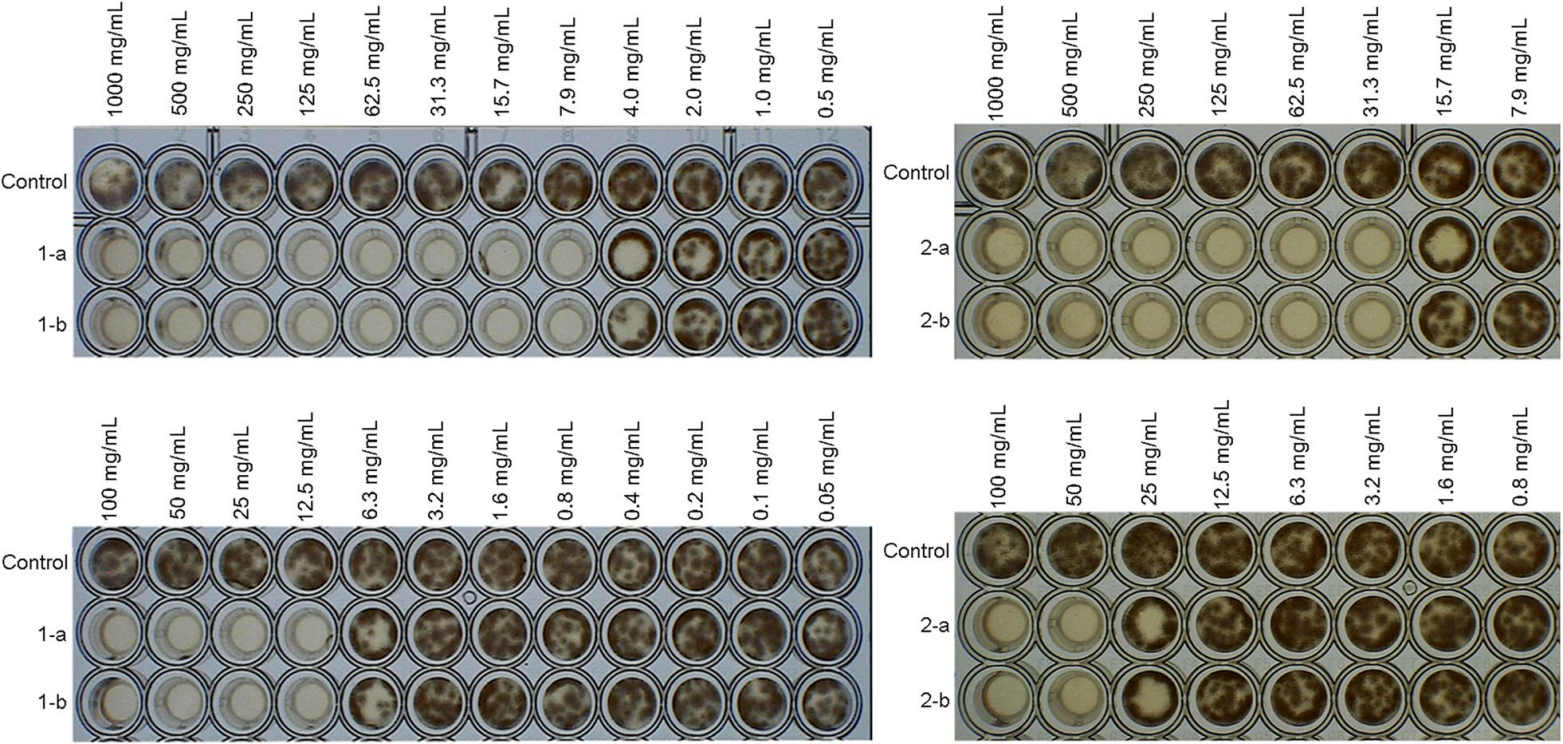
MIC evaluation (Micro dilution assay) of the purified active components **1** and **2** in *A. flavus*

The MIC values of compounds **1** and **2** were evaluated in a 96 microwell plate against the selected test fungi (Table 2). The organic extract (Mix) and Myconazole were used as references (Table 2). The isolated compounds showed higher antifungal activity than the whole organic extract, with the only exception for **2** in the case of *P. griseofulvum*. The most sensitive species was *A. flavus* (Fig. 5) with a MIC value of 7.9 µg/mL and 31.3 µg/mL for **1** and **2**, compound **1** being the most active derivative. A similar behaviour was observed for *P. griseofulvum*. By contrast, *T. pleuroticola* showed to be definitely more sensitive to **2** (MIC 125 µg/mL) than to **1** (> 500 µg/mL). In addition, a significant growth reduction was observed at concentrations much lower than MIC (Table 2). In all cases the activity was fungistatic, while no fungicidal action was recorded as demonstrated by subsequent culturing of the treated fungi. Moreover, microscopic observations showed that conidial germinations of the tested fungi were not completely inhibited by the extract, which acted mainly blocking the hyphal growth.

**Table 2:** Minimal inhibitory concentration (MIC) and growth reduction (GR) values of the organic extract (Mix) and of compounds **1** and **2**

	*P. griseofulvum*		*A. flavus*		*T. pleuroticola*	
	MIC	GR	MIC	GR	MIC	GR
Mix	100	25	62.5	31.3	> 500	125
1	25	7.9	7.9	4.0	500	250
2	100	25	31.3	15.7	125	50
Control	2	–	0.5	–	1	–

## Conclusions

The marine strain IG100, cultivated in appropriate conditions (saline medium, NaCl 3.5%), produced significant amounts of two bioactive terpenoids, terperstacin **1** and 19-acetyl-4-hydroxydictyodiol **2** (9.1 mg and 8.9 mg, respectively from 55 mg of EtOAc extract). The two compounds showed significant antifungal activities. Compound **1** was characterized by higher activity on *A. flavus* (MIC 7.9 µg/mL) and *P. griseofulvum* (MIC 25 µg/mL) compared to compound **2** (MIC 31.3 and 100 µg/mL, respectively). On the other hand, the latter showed higher antifungal activity against *T. pleuroticola* (MIC 125 µg/mL) than **1** (MIC 500 µg/mL). Both compounds showed a significant antifungal activity against three toxinogenic/pathogenic fungal species representing genera  with large number of species and great significance in ecological, agronomical, economical and clinical fields. It is worth noting that compounds **1** and **2**, produced by the epiphytic fungus *M. humicola*, could exert for the host (i.e. *Posidonia oceanica*) a specific defensive role, in accordance with the general behaviour reported for xenicanes produced by brown algae [52].

## Materials and methods

### Microorganisms

The strain IG100 was isolated from the marine phanerogam *Posidonia oceanica* in the Tyrrhenian Sea and cryogenically maintained at − 40 °C in the culture collection of microorganisms of the “Laboratorio di Ecologia dei Funghi Marini”, DEB (University of Tuscia, Viterbo, Italy). The test strains (*Aspergillus flavus* IG133, *Trichoderma pleuroticola* IG137, *Penicillium griseofulvum* TSF04, *Bacillus pumilus* KB66 and *Escherichia coli* JM109) were from the same culture collection. Strains had been revitalized and sub-cultured on Malt Extract Agar Seawater (MEAs) medium (50 g MEA – Sigma -Aldrich dissolved in 1 L of filtered seawater) and Luria Bertani Seawater (LBs) broth (25 g LB – Sigma -Aldrich dissolved in 1 L of filtered seawater) for the fungal and bacterial strains, respectively.

### Phylogenetic analysis

Strain IG100 was previously attributed by ITS and β-tubulin (tub2) gene analyses to the species *Mariannaea humicola* [30].

The phylogenetic analysis was carried out on β-tubulin (tub2) gene, partial cds utilizing the sequences present in the NCBI (National Center for Biotechnology Information advances science and health by providing access to biomedical and genomic information) for the *Mariannaea* species. A Bayesian Inference was performed [57]. For this analysis, the best fitting evolution model was obtained using the jModeltest software [58] under 88 possible substitution models. The model, obtained using the Akaike Information Criterion (AIC), was TIM1ef, G = 0.430 [59]. The Bayesian Posterior Probability analysis (BPP) was carried out using the Markov Chain Monte Carlo algorithm with generations number = 1,000,000, sub-sampling frequency = 100 and a burn-in fraction = 0.25. BPP values are reported in the resulting tree.

### Preliminary screening

The preliminary screening for antifungal and antibacterial activity was carried out with the agar plug diffusion method and agar well diffusion method, this last to evaluate the activity of the compounds in liquid condition [36]. All tests were carried out on saline media and test organisms were selected among marine and/or salt tolerant strains to avoid interferences related to salt presence.

The halotolerant strains of *Bacillus pumilus* KB66, *Escherichia coli* JM109 and *Penicillium griseofulvum* TSF04 were used as test organisms.

To produce standardized inoculum the bacteria were grown in LBs for 24 h at 30 °C in an orbital shaker at 150 rpm and the fungus in MEAs for 7 days at 25 °C. Conidia suspensions were prepared in sterile filtered seawater supplemented with 0.01% of Tween 80 and diluted to obtain a final inoculum ranging from 0.5 × 10^5^ to 1.0 × 10^5^. Test plates used in the following tests were inoculated by spreading 100 µL of bacteria culture and 300 µL of spore suspension.

#### Plug diffusion method

* Mariannaea humicola* IG100 was cultured for 7 days on MEAs medium supplemented with 0.2 g/L streptomycin sulfate at 25 °C; agar plots were aseptically cut near the front of the colony and deposited on the agar surface of plates previously inoculated by test organisms. After incubation (24 h, 30 °C for bacteria and 48–72 h, 25 °C for fungus) the antimicrobial activity was estimated measuring the inhibitions area (halos).

#### Well diffusion method

Strain IG100 was cultured in Malt Extract Seawater (MEs) [30]. Five Erlenmeyer flask (1000 mL), containing 250 mL of medium, were inoculated with 1 mg/mL dry weight of mycelium grown for 5 days on MEAs and incubated for 12 days at 25 °C on a rotary shaker (150 rpm). Subsequently, one Erlenmeyer flask culture was aseptically filtered to separate the broth from the biomass; the others cultures were directly extracted with EtOAc (1 L x 3 times). The EtOAc extract solution was concentrated to dryness by a rotary evaporator and utilized as crude extract. In each inoculated test plate, four holes (6–8 mm) were punched aseptically with a sterile cork borer. 100 µL of broth or EtOAc extract at 0.6 mg/mL, 2% DMSO, were added in two of the holes. While the remaining two holes were used for a positive (Streptomycin 0.2 mg/mL for bacteria and Myconazole 0.05 mg/mL for fungal strain) and negative control (culture media). Plates were incubated as reported above, the inhibition zone around the wells were measured and the antimicrobial activity was estimated as:

1 $${\text{Antimicrobial activity }}\left( \% \right) \, = \, \left( {{\text{IC}} - {\text{IS}}} \right)/{\text{IC }} \times { 1}00$$

where, IC is the inhibition area in positive well and IS the inhibition area in well containing potential antimicrobial agent. Each analysis was carried out in triplicate.

### Antifungal activity assay

The biological activities of EtOAc extract and its principal compounds were tested in a liquid growth medium (MBs) against *P. griseofulvum* utilizing the broth dilution methods. Compounds were first dissolved in DMSO, which was then diluted to a 2% aqueous solution to obtain each compound concentration of 5 × 10^− 3^ M. The compounds were added, in a 24-well microplate, to the suspension of the *P. griseofulvum* spore (1 × 10^5^ m/L) to obtain final concentrations of 1, 10, 100, 1000 µg/mL. The conidial suspension was prepared in MEBs as reported above. DMSO at appropriate concentrations was used as control treatments.

MIC values were determined for the crude EtOAc extract and active fractions against the following fungal strains: *A. flavus* IG133, *P. griseofulvum* TSF04 and *T. pleuroticola* IG137. The broth micro-dilution methods in a 96-well microplate was utilized; twofold dilutions of the antifungal agents, Myconazole as positive control and a negative control with DMSO (concentrations < 2%) were made. The test was carried out in duplicate utilizing two initial concentrations and the relative twofold dilutions: S1, 1000–1 µg/mL and S2, 100 –0.1 µg/mL. Spore suspensions of the test organisms were standardized to the final concentrations of 0.5 × 10^5^ conidia m/L. The 96-well microplates were incubated for 48 h at 25 °C.

For the determination of MIC endpoint, a viewing device (Canon visualizer RE450) was utilized.

Fungistatic or fungicidal activities were determined by subcultures in MEAs 20 µL of the spore suspensions taken from the MIC wells.

### Isolation and characterization of bioactive secondary metabolites—chemistry general

Reagents and solvents were obtained from commercial suppliers (Sigma-Aldrich Srl, Milan, Italy) and used without further purification. TLC chromatography was performed on precoated aluminium silica gel SIL G/UV254 plates (Macherey-Nagel & Co.). The detection occurred by UV lamp (254 nm). Merck silica gel 60 was used for flash chromatography (23–400 mesh). All products were dried in high-vacuum (10 –3 mbar). ^1^H NMR and ^13^C NMR spectra were measured on a Bruker Avance DRX400 (400 MHz/100 MHz) spectrometer using TMS as internal standard. Chemical shifts in ^1^H NMR spectra are reported in parts per million (ppm) on the δ scale from an internal standard of residual CDCl_3_ (7.28 ppm). Data are reported as follows: chemical shift, multiplicity (s = singlet, d = doublet, t = triplet, q = quartet, m = multiplet, br = broad), coupling constant in hertz (Hz), and integration. Chemical shifts of ^13^C NMR spectra are reported in ppm from the central peak of CDCl_3_ (77.23 ppm). HRESIMS spectra were recorded with a Thermo Scientific Q Exactive hybrid quadrupole-Orbitrap mass spectrometer. Infrared (IR) spectra were recorded on a Perkin-Elmer 2000 FT-IR. A semipreparative UHPLC Thermo Scientific Dionex Ultimate 3000 equipped with multi-wave length detector was used for purification. The column used was a Thermo Scientific Hypersil GOLD (15.0 cm length, 4.6 mm I.D., 3 µm particle size) eluted with mixtures of water containing 0.05% formic acid (HCOOH; solvent A) and acetonitrile (solvent B) at a flow rate of 0.5 mL/min. Detection was at 254 nm and runtime was set at 60 min.

The ethyl acetate extract was concentrated in vacuo. The dry residue (55 mg) was purified by repeated cycles of silica gel chromatography using dichloromethane/methanol (98:2) as eluent. Five major fractions (PO1–PO5) were isolated from the overall EtOAc extract (5.9, 10.5, 9.5, 14.6, and 9.5 mg, respectively). Semipreparative UHPLC was performed to further purify the fractions endowed with the higher biological activity (PO3 and PO5) obtaining 9.1 mg of compound **1** and 8.9 mg of compound **2**.

Terpestacin **1**: pale-yellow oil; [α]_D_ = − 21 (c 0.1, MeOH); IR (neat) 3365, 2932, 1699, 1653/cm; ^1^H NMR (400 MHz, CDCl_3_) δ 5.79 (s, 1H), 5.41 (m, 1H), 5.25 (dd, *J* = 10.1, 5.2 Hz, 1H), 5.14 (m, 1H), 4.07 (dd, *J* = 10.1, 4.0 Hz, 1H), 3.90 (dd, *J* = 10.4, 7.0 Hz, 1H), 3.83 (dd, *J* = 10.4, 5.5 Hz, 1H), 2.72 (dd, *J* = 11.3, 2.1 Hz, 1H), 2.68 (m, 1H), 2.45 (d, *J* = 17.4 Hz, 1H), 2.40 (dd, *J* = 13.7, 10.4 Hz, 1H), 2.22–2.22 (m, 2H), 2.12–2.12 (m, 2H), 2.04–1.90 (m, 2H), 1.80–1.68 (m, 3H), 1.65 (s, 3H), 1.64 (s, 3H), 1.58 (s, 3H), 1.30 (d, *J* = 7.3 Hz, 3H), 1.01 (s, 3H); ^13^C NMR (100 MHz, CDCl_3_) δ 208.0, 149.0, 146.8, 138.3, 136.7, 133.1, 129.1, 124.5, 121.7, 76.7, 66.3, 49.8, 49.1, 40.5, 39.5, 37.3, 35.1, 30.0, 29.0, 24.0, 16.4, 15.8, 15.5, 14.6, 10.7. ppm; HRESIMS *m/z* [M +Na]^+^ calcd. for C_25_H_38_O_4_Na 425.2662, found 425.2682.

19-acetyl-4-hydroxydictyodiol **2**: colorless oil; [α]_D_ = − 43 (c 0.02, CHCl_3_); IR (neat) 3439, 2921, 1737, 1638, 1455, 1381 cm^− 1^; NMR ^1^H (CDCl_3_, 400 MHz) δ 5.82 (dd, *J* = 8.4, 3.0 Hz, 1H), 5.30 (d, *J* = 11.4 Hz, 1H), 5.07 (t, *J* = 6.6 Hz, 1H), 4.47 (d, *J* = 12.6 Hz, 1H), 4.39 (d, *J* = 12.6, Hz, 1H), 4.28 (br, 1H), 3.81 (t, *J* = 10.2 Hz, 1H), 3.55 (dd, *J* = 10.2, 4.2 Hz, 1H), 3.14 (dd, *J* = 9.6, 4.2 Hz, 1H), 3.11 (m, 1H), 2.63 (dd, *J* = 14.4, 9.0 Hz, 1H), 2.37 (d, *J* = 12.6, 1H), 2.14 (dd, *J* = 12.6, 3.0 Hz, 1H), 2.09 (br, 1H), 2.06 (s, 3H), 2.00 (m, 1H), 1.97 (s, 3H), 1.94 (m, 1H), 1.83 (m, 1H), 1.68 (s, 3H), 1.60 (s, 3H), 1.30 (m, 1H), 1.21 (m, 1H), 1.02 (d, *J* = 6.6, 3H) ppm; ^13^C (CDCl_3_, 100 MHz) δ 170.7, 142.4, 135.2, 131.9, 130.0, 128.5, 124.5, 74.6, 67.0, 60.8, 51.4, 49.5, 41.5, 38.7, 33.3, 28.2, 26.2, 25.8, 21.2, 20.4, 17.9, 16.4 ppm; HRESIMS *m/z* [M+Na]^+^ calcd. for C_22_H_36_O_4_Na, 387.2511, found 387.2508.

## Data Availability

The authors promise the availability of supporting data.
